# Drainage in Ovariotomy

**Published:** 1874-09

**Authors:** 


Drainage in Ovariotomy.— * * * It seemed to be a suitable case, if any such could be found, for the insertion of a drainage-tube through the retro-uterine *cul-de-sac;* and, with the concurrence of his colleagues, Dr. Savage and Mr. Ross Jordon, Mr. Tait passed a double loop of large-sized wire drainage-tube through the roof of the vagina.

A few hours after the operation, a faintly reddish-colored serum began to pass from the wound about its middle, and continued to do so up to the patient's death; probably several pints in all so passed. Not a drop, however, came by the tube, until the body was moved after death. At the post-mortem examination it was evident that the tube was occluded by the pressure of the intestines; for as soon as they were lifted out of the pelvis a large quantity of fluid flowed rapidly through the tube.

The patient died of shock; for there had been no hemorrhage, nor were there any signs of peritonitis.

Of five other cases of death after ovariotomy, in which Mr. Tait has obtained or seen post-mortem examinations, and in which the cause of death was the effusion of purulent or other fluid into the peritoneum, in not one could a drainage-tube have been of the slightest use; and the possibility of benefit arising from the insertion of one seems to be very remote, while the probability of its doing harm is very considerable.—*The London Lancet—May.*

